# The effectiveness of transcranial magnetic stimulation in ameliorating limb motor function disorders after stroke: an umbrella review

**DOI:** 10.3389/fneur.2026.1741500

**Published:** 2026-03-23

**Authors:** Xiaoduo Yao, Meiyi Luo, Wenping Zhang, Yue Shen, Luye Feng, Chunting Liang, Dehua Wang, Xiaofei Cheng, Jiqin Tang

**Affiliations:** 1 School of Rehabilitation, Shandong Second Medical University, Weifang, China; 2 Yixin Street Community Health Service Center in Shuangliu District, Chengdu, China; 3 School of Rehabilitation, Shandong University Of Traditional Chinese Medicine, Jinan, China

**Keywords:** cerebrovascular disease, stroke, transcranial magnetic stimulation, umbrella review, upper limb dysfunction

## Abstract

**Objective:**

This study aimed to reassess systematic reviews and meta-analyses investigating the effects of transcranial magnetic stimulation (TMS) on limb motor impairment in stroke patients.

**Methods:**

The PRISMA 2020 checklist and AMSTAR 2 tool were used to evaluate the completeness of reporting and methodological quality of the included literature. The GRADE system was used to assess the quality of evidence for the outcome measures reported in the included systematic reviews and meta-analyses.

**Results:**

A total of 34 articles were included. PRISMA 2020 scores ranged from 17.5 to 26, with a mean score of 22.44. Based on the AMSTAR 2 methodological quality assessment, two studies were rated as high quality, one as moderate, six as low, and 25 as very low. According to the GRADE system, the evidence consisted of four high-quality, 29 moderate-quality, 42 low-quality, and 46 very low-quality evidence bodies.

**Conclusion:**

The findings suggest that TMS has a positive impact on the recovery of limb motor function in stroke patients, with notable improvements observed in upper limb function (e.g., ARAT and FMA-UE scores), walking ability, and activities of daily living. However, the overall strength of these conclusions is constrained by the generally low methodological quality of the included literature.

## 1 Introduction

Stroke, also referred to cerebrovascular accident, is a type of brain tissue damage disease caused by the sudden rupture or blockage of blood vessels in the brain, leading to insufficient blood supply to the brain (1). Globally, it has emerged as a leading cause of disability and death (2). In China, the incidence rate of stroke is high, making the rehabilitation of stroke patients a significant concern (3). Most stroke survivors experience limb motor impairment, with more than half facing long-term deficits (4). Currently, there are numerous rehabilitation approaches for post-stroke limb motor function impairments, including biofeedback therapy, mirror therapy, acupuncture, virtual reality, and neuromuscular electrical stimulation (5). However, the efficacy of these interventions requires further validation (6).

Since its introduction by Barker et al. (59), transcranial magnetic stimulation (TMS) has been widely studied and shown promising effects in improving motor function in stroke patients (7, 8). Based on the interhemispheric competition model, low-frequency TMS is commonly applied to the lesioned hemisphere, while high-frequency TMS is administered to the contralateral hemisphere, aiming to rebalance cortical excitability and promote motor recovery (9).

Although several systematic reviews and meta-analyses have been published on TMS for post-stroke motor recovery, inconsistencies remain in outcome measures, result quality, and conclusions. This study aims to reassess the reporting quality (using PRISMA 2020), methodological rigor (using AMSTAR 2), and evidence quality (using the GRADE system) of the included articles, to further synthesize existing evidence, and to offer clinical decision support and treatment guidance for the application of TMS in treating motor dysfunction in stroke patients. The study protocol has been registered with the International Prospective Systematic Review Registry (PROSPERO), ID: CRD42024615524.

## 2 Data and methods

### 2.1 Data sources and search strategy

The literature search was performed across four electronic databases (PubMed, Web of Science, Embase, and the Cochrane Library) up to June 20, 2024. To ensure a thorough search, reference lists of included articles were reviewed for additional relevant studies. The search strategy combined both subject headings and free-text terms, including “transcranial magnetic stimulation”, “stroke”, “motor function”, “dyskinesias”, “upper extremity”, “lower extremity”, “systematic review”,and “Meta-Analysis” as topics. The search strategy was appropriately adjusted based on the specific subject headings of each database.

### 2.2 Inclusion and exclusion criteria

The inclusion criteria were as follows: ① Participants: Patients with a confirmed diagnosis of post-stroke motor dysfunction in the limbs, without other severe complications; ② Interventions: The experimental group received TMS, either as a standalone treatment or in combination with conventional rehabilitation therapy; ③ Comparator: The control group received sham stimulation, either alone or in combination with other conventional rehabilitation treatments; ④ Primary outcomes: hand function score, upper limb function score, lower limb function score, limb function score, spasticity rating scale, balance function score, walking ability score, daily living ability score, changes in cortical excitability, and stroke severity. Secondary outcomes included adverse reactions. Included studies had to report at least one primary outcome; ⑤ Study type: systematic reviews or meta-analyses of randomized controlled trials (RCTs).

The exclusion criteria were as follows: ① Non-English publications; ② Articles in the form of conference abstracts, narrative reviews, or case reports; ③ Studies with outcome measures that did not meet the predefined criteria.

### 2.3 Study selection and data extraction

Prior to the screening and quality assessment phases, all reviewers completed a standardized training session on the methodology of systematic reviews and meta-analyses. The training, led by an experienced researcher with a proven record of publishing meta-analyses in related fields, covered the detailed application of relevant assessment tools—including risk of bias, evidence quality grading, and study eligibility criteria—through standardized definitions, operational steps, and practical examples.

Following the training, all reviewers completed a knowledge test with standardized questions to ensure a consistent understanding of the methodology. Those who passed were approved to conduct independent evaluations.

To ensure the reliability of the evaluation, inter-rater reliability testing was performed during the initial stage of screening and grading. Disagreements were resolved through discussion or, when necessary, by a third senior reviewer to reach consensus. This process ensured high consistency among all raters throughout the article review process.

Two authors independently screened the literature, using EndNote software to remove duplicate articles. A preliminary selection was made by reviewing the titles and abstracts of the articles, followed by an in-depth reading for final selection, ensuring that the included articles met the predefined criteria and requirements. In case of disagreements during the literature screening, a third author made the decision.

The same two authors independently extracted data from the included articles using a predefined form, capturing information such as the first author, number of studies included, publication year of the original studies, intervention measures, stimulation sites, outcome indicators, methodological quality assessment tools used, whether GRADE evaluation was conducted, main conclusions, etc. All extracted data were cross-checked and tabulated.

### 2.4 Quality assessment of reporting

The reporting quality for the included studies was assessed using the 27-item checklist from PRISMA 2020 (10). Each item was scored as follows: not reported (0 points), partially reported (0.5 points), or fully reported (1 point), with a maximum total score of 27 points. A score of 21 to 27 points indicated relatively complete reporting, a score of 15 to 21 points indicated moderate deficiencies in reporting, and a score below 15 points indicated significant information gaps.

### 2.5 Methodological quality assessment

The methodological quality of the included studies was appraised with the AMSTAR 2 checklist, which contains 16 items, each judged as “yes,” “no,” or “partial yes” (11). The methodological quality was categorized into four levels: high, moderate, low, and very low. Items 2, 4, 7, 9, 11, 13, and 15 were considered critical. The rating was determined primarily by the number of unmet critical items. A study was rated as high quality if it had no more than one non-critical item unmet; moderate if more than one non-critical item was unmet; low if one critical item was unmet; and very low if multiple critical items were unmet.

### 2.6 Evidence quality assessment of outcome measures

The evidence quality of the outcome measures in the included studies was evaluated using the GRADE system, with four levels of quality: high, moderate, low, and very low (12). The initial quality level for RCTs is high. Five downgrading factors were considered: risk of bias, inconsistency, indirectness, imprecision, and publication bias. After one, two, or three levels of downgrading, the evidence quality levels are, respectively, moderate, low, and very low.

## 3 Results

### 3.1 Screening process and results

**Figure 1:**
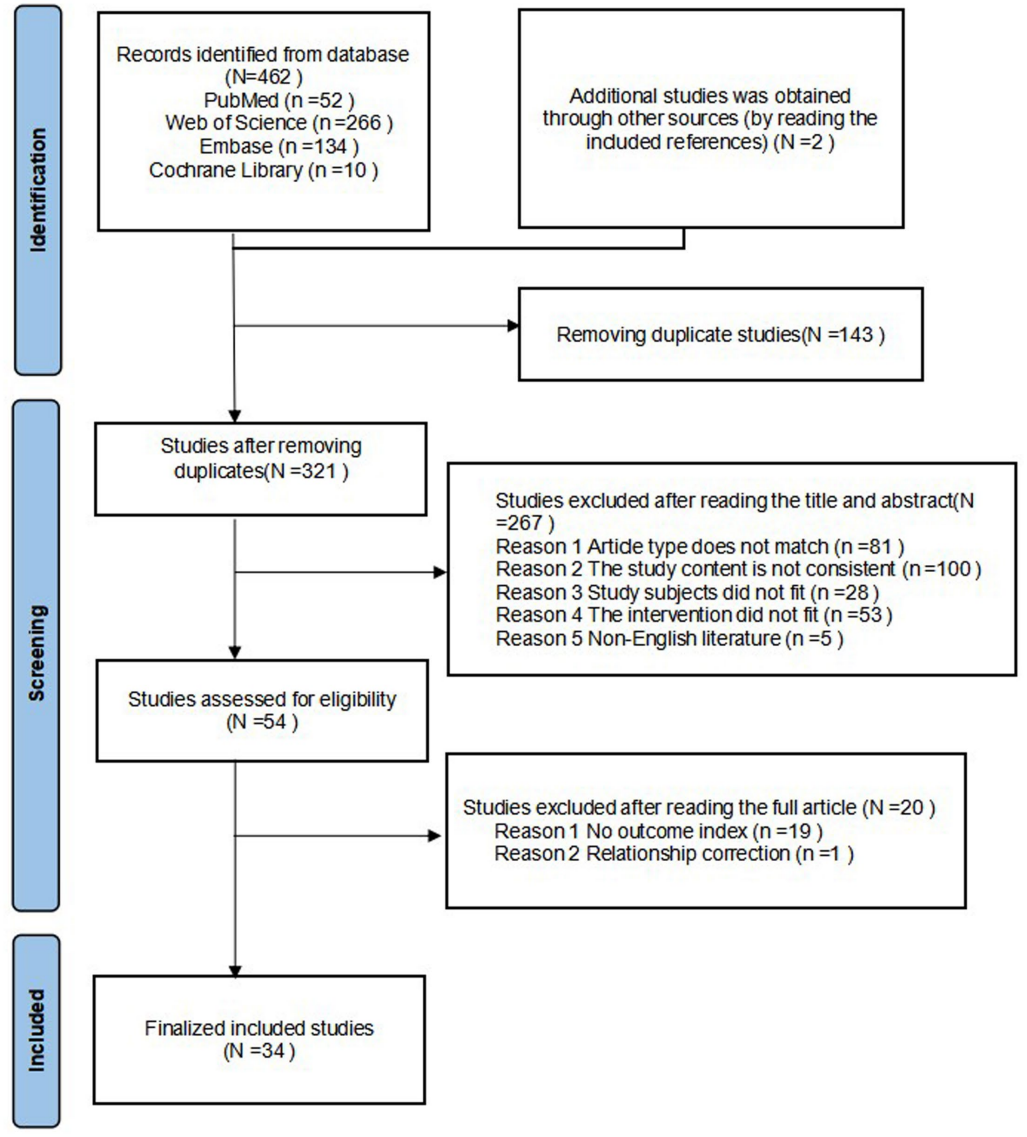
Flow chart of literature screening.

A total of 464 articles were retrieved. After removing 143 duplicate articles and screening the titles and abstracts, an additional 267 articles were excluded. Following a full-text assessment of the remaining 54 articles, 20 were excluded for not meeting the eligibility criteria, resulting in 34 studies included in the final analysis (13–46). The literature screening process is depicted in Figure 1.

### 3.2 Characteristics of included studies

Among the 34 included studies, two involved child samples (19, 39), and 14 reported adverse reactions. Sixteen studies applied the Cochrane Risk of Bias tool to assess the methodological quality of primary studies. The specific characteristics of the included studies are detailed in Supplementary Table 1.

### 3.3 Evaluation results of the included literature

#### 3.3.1 Report quality evaluation

The results are shown in Supplementary Table 2. The scores of the included studies ranged from 17.5 to 26, indicating a relatively high level of reporting completeness. Only two studies had a completeness level below 70%. Twenty-seven studies were classified as having relatively complete reports, while seven exhibited certain deficiencies. None of the studies fully satisfied all PRISMA 2020 checklist criteria. Key reporting shortcomings included the absence of registration information in 49.8% of the studies, lack of an assessment of the credibility of the body of evidence in 79.4%, and incomplete search strategies in 68.6% of cases. These omissions contributed to limitations in the overall reporting quality.

#### 3.3.2 Methodological quality assessment

The methodological quality of the included studies, evaluated using the AMSTAR 2 tool was generally low. Among the studies, two were rated as high quality, one as moderate, six as low, and twenty-five as very low quality. With respect to critical domains, 20 studies failed to register their protocols, 18 did not discuss the impact of risk of bias on the results, and only nine examined the potential influence of publication bias. For non-critical items, ten studies did not specify whether literature selection was performed independently by two reviewers, seven did not describe independent data extraction by two persons, only one reported the funding sources of the included studies, four did not address potential bias risks, eleven failed to provide adequate explanations for heterogeneity, and eight either did not report or partially reported conflicts of interest and funding information. The AMSTAR 2 methodological quality assessment is detailed in Supplementary Table 3. The results of the PRISMA 2020 and AMSTAR 2 assessments are presented together in Table 1.

**Table 1:** PRISMA grade and AMSTAR2 methodological quality evaluation grade.

Included in the literature	PRISMA grade	Amstar2 methodological quality evaluation grade
Chen et al. (13)	24	Low
Chen et al. (14)	26	Very low
Chen et al. (15)	25	Very low
Gao et al. (16)	23.5	Very low
Ghayour-Najafabadi et al. (17)	24.5	Low
Graef et al. (18)	19.5	Very low
Hao 2013 (19)	21	Very low
He et al. (20)	24.5	Medium
Hsu et al. (21)	19.5	Very low
Huang et al. (22)	24.5	High
Jiang et al. (23)	26	High
Kang et al. (24)	22	Very low
Le et al. (25)	22	Very low
Li et al. (26)	24	Very low
Li et al. (27)	19.5	Very low
Liu et al. (28)	22	Very low
McIntyre et al. (29)	21	Very low
Narayan et al. (30)	18	Very low
Ni et al. (31)	17.5	Very low
Tang et al. (32)	24	Low
Tian et al. (33)	21.5	Low
Tung et al. (34)	23	Very low
van Lieshout et al. (46)	23	Low
Vaz et al. (35)	24.5	Very low
Veldema et al. (36)	21.5	Very low
Wang et al. (37)	22.5	Very low
Wang et al. (38)	21.5	Very low
Xiang et al. (39)	23	Low
Xu et al. (40)	20.5	Very low
Zeng et al. (41)	24	Very low
Zhang et al. (42)	19.5	Very low
Zhang et al. (43)	21.5	Very low
Zhang et al. (44)	24	Very low
Zhang et al. (45)	24	Very low

### 3.4 Evidence quality assessment

The GRADE system was used to assess the evidence quality of 121 bodies of evidence across 13 outcome indicators. Among these, four bodies of evidence were rated as high quality, 29 as moderate quality, 42 as low quality, and 46 as very low quality. The results are presented in Table 2. The overall low quality of the evidence bodies is primarily attributed to bias risk and publication bias, as detailed in Supplementary Table 4.

**Table 2:** GRADE quality of evidence.

Outcome measures and the included studies	Number of studies on outcome measures	Evidence grading
① Hand flexibility		
Chen et al. (13)	8	Middle
Chen et al. (14)	6	Middle
Graef et al. (18)	2	Very low
Tang et al. (32)	6	Middle
He et al. (20)	3	Low
Jiang et al. (23)	4	High
Le et al. (26)	4	Middle
Le et al. (25) (Hand function)	3	Middle
Narayan et al. (30)	2	Low
van Lieshout et al. (46)	28	Very low
Zhang et al. (43) (short-term)	27	Middle
Zhang et al. (43) (long-term)	11	Middle
Zhang et al. (44)	6	Middle
② Hand strength		
He et al. (20)	5	Middle
Tang et al. (32)	5	Low
Zhang et al. (44)	11	Middle
③ Upper limb functional activity		
van Lieshout et al. (46)	20	Low
Zhang et al. (42)	14	Low
Gao et al. (16)	2	Very low
Graef et al. (18)	4	Very low
Graef et al. (18)	3	Very low
Chen et al. (15)	5	Very low
Gao et al. (16)	5	Very low
Graef et al. (18)	3	Very low
Jiang et al. (23)	5	High
Li et al. (26)	5	Very low
Chen et al. (14)	15	Low
Hao 2013 (19)	4	Very low
Hsu et al. (21)	17	Low
Zhang et al. (44)	10	Middle
④ The Fugl-Meyer upper limb score		
Chen et al. (13)	39	Middle
Chen et al. (15)	6	Low
Gao et al. (16)	6	Low
Graef et al. (18)	4	Low
He et al. (20)	15	Low
Huang et al. (22)	8	Low
Jiang et al. (23)	8	High
Li et al. (26)	14	Low
Tang et al. (32)	11	Low
van Lieshout et al. (46)	15	Very low
Zhang et al. (44)	7	Low
Zhang et al. (42)	12	Very low
⑤ Lower limb motor function		
Chen et al. (14)	3	Low
Gao et al. (16)	4	Very low
Ghayour-Najafabadi et al. (17)	6	Low
He et al. (20)	6	Middle
Jiang et al. (23)	4	Middle
Li et al. (27)	3	Low
Liu et al. (28)	7	Very low
Tung et al. (34)	4	Low
Zeng et al. (41)	4	Middle
Zhang et al. (45)	9	Low
Ni et al. (31)	8	Very low
⑥ Body motor function		
Gao et al. (16)	10	Very low
He et al. (20)	21	Middle
Xiang et al. (39)	42	Middle
⑦ Convulsion		
Chen et al. (14)	7	Middle
Chen et al. (15)	5	Very low
Gao et al. (16)	4	Very low
Graef et al. (18)	2	Very low
Jiang et al. (23)	5	Middle
Liu et al. (28)	5	Low
McIntyre et al. (29)	6	Very low
Wang et al. (38)	11	Low
Xu et al. (40)	5	Very low
⑧ balanced capacity		
Chen et al. (14)	3	Low
Ghayour-Najafabadi et al. (17)	3	Low
Liu et al. (28)	2	Very low
Wang et al. (37)	4	Middle
Gao et al. (16)	2	Low
Jiang et al. (23)	3	Low
Wang et al. (37)	8	Low
Zeng et al. (41)	8	Very low
Zhang et al. (45)	4	Very low
Kang et al. (24)	9	Low
Li et al. (27)	3	Very low
Ghayour-Najafabadi et al. (17)	5	Low
Ni et al. (31)	5	Very low
Wang et al. (37)	3	Middle
Wang et al. (37)	2	Middle
Wang et al. (37)	3	Middle
⑨Walking ability		
Chen et al. (14)	6	Middle
Ni et al. (31)	6	Very low
Tung et al. (34)	3	Low
Vaz et al. (35)	3	Low
Li et al. (27)	6	Low
Veldema et al. (36)	9	Middle
⑩Ability of daily living activities		
Chen et al. (14)	9	Middle
Huang et al. (22)	3	Low
Liu et al. (28)	2	Very low
Gao et al. (16)	3	Low
Hao 2013 (19)	2	Very low
Li et al. (26)	6	Very low
Zhang et al. (45)	8	Very low
Xiang et al. (39)	7	Middle
He et al. (20)	11	Low
Wang et al. (37)	6	Low
⑪Motion evoked potential		
Chen et al. (15) (affected side)	3	Very low
Chen et al. (15) (unaffected side)	2	Very low
Huang et al. (22) (affected side)	1	Very low
Huang et al. (22) (unaffected side)	3	Very low
Huang et al. (22)	4	Very low
Le et al. (25) (affected side)	3	Very low
Li et al. (27) (affected side)	2	Very low
Li et al. (27) (unaffected side)	1	Very low
Tang et al. (32) (affected side)	3	Low
Tang et al. (32) (unaffected side)	2	Low
Tung et al. (34)	3	Low
Zhang et al. (44) (affected side)	4	Middle
Zhang et al. (44) (unaffected side)	8	Middle
Zhang et al. (45) (affected side)	5	Very low
⑫Motion threshold		
Hsu et al. (21) (affected side)	5	Very low
Le et al. (25)	3	High
Huang et al. (22) (affected side)	2	Very low
Huang et al. (22) (unaffected side)	4	Very low
Huang et al. (22)	6	Very low
Zhang et al. (44) (affected side)	4	Very low
Zhang et al. (44) (unaffected side)	6	Low
⑬Stroke severity		
He et al. (20)	7	Low
Tian et al. (33)	2	Low
Li et al. (26)	6	Very low

### 3.5 Outcome indicator results

Among the included studies, 16 analyzed the therapeutic effects on hand function. Four studies specifically evaluated performance on box-and-block test (BBT) (18), nine-hole peg test (NHPT) (23), Jebsen-Taylor Hand Function Test (JTHFT) (20), and overall hand function (25). Results consistently indicated that TMS did not significantly improve finger dexterity. However, three studies all showed positive effects on the recovery of hand strength (20, 32, 44).

Fourteen studies analyzed the therapeutic effects on upper limb functional activities. Seven of these studies indicated that TMS has a clinically positive impact on the recovery of motor function in the affected upper limbs of stroke patients, particularly as measured by the Action Research Arm Test (ARAT) scores (15, 16, 23). Additionally, there was a positive influence on upper limb flexibility (44). In contrast, seven studies showed no significant therapeutic effect on the recovery of upper limb motor function. The results for the Wolf Motor Function Test (WMFT) were not significantly affected (16, 18). In this manuscript, Li et al. (26) mentioned that inhibitory rTMS had no effect on ARAT scores, while excitatory rTMS significantly altered ARAT scores.

Twelve studies independently analyzed the Fugl-Meyer Assessment of Upper Extremity (FMA-UE) scores in patients. Ten studies demonstrated that TMS is effective in improving FMA-UE scores, with intermittent theta burst stimulation (iTBS) showing the most significant improvement during the acute phase post-intervention (22). Two studies indicated no therapeutic effect on improving FMA-UE scores.

Eleven studies assessed lower limb motor function, with five reporting no improvement. With the exception of Chen et al. (14), who used FMA-Lower Extremity (FMA-LE) and a lower limb motor index, and Ni et al. (31), who did not provide detailed outcomes, all studies used FMA-LE as the primary outcome measure.

Three studies analyzed the therapeutic effects on overall limb motor function, and all results indicated that TMS has a positive impact on the recovery of limb motor function.

Nine studies analyzed the therapeutic effects on spasticity. Three studies reported no significant improvement in Modified Ashworth Scale (MAS) scores for patients with spasticity, and one study indicated that iTBS did not significantly improve MAS scores (16).

Sixteen studies analyzed evaluated balance ability. Seven of these studies showed no significant improvement. One study employed multiple methods to assess balance ability and demonstrated that cerebellar TMS significantly improved the Timed “Up & Go” Test (TUG), Berg Balance Scale (BBS), and post-intervention stability index (SI) with eyes open and closed, but did not significantly improve walking time in the 10-Meter Walk Test (10MWT) at the end of the follow-up period (37).

Six studies assessed gait function, five of which reported significant improvements. Veldema et al. (36) combined results for gait, balance, and lower limb motor function, and post-intervention data suggested that rTMS had a minimal effect on gait, balance, and lower limb motor function in stroke patients.

Ten studies analyzed the therapeutic effects on activities of daily living (ADL) and used either the Modified Barthel Index (MBI) or the Barthel Index (BI) for evaluation. Two of these studies showed no significant effect on BI scores.

Fourteen studies analyzed motor-evoked potentials (MEPs), six of which found no significant effects. Additionally, seven studies examined the impact on motor thresholds, with three indicating no significant effect. In this manuscript, Huang et al. (22) demonstrated that iTBS increased cortical excitability on the unaffected hemisphere while reducing it on the affected side.

Three studies evaluated changes in stroke severity. In this manuscript, Tian et al. (33) indicated that rTMS had no significant effect on National Institute of Health stroke scale (NIHSS) scores, whereas He et al. (20) observed significant improvements. Li et al. (26) demonstrated behavioral improvements in the Brunnstrom stages of recovery.

## 4 Discussion

### 4.1 The completeness of the report, the quality of the methodology and the quality of the evidence need to be improved

To improve the reporting completeness and methodological rigor of future research, authors should ensure study registration details are provided, reasons for study exclusions are clearly stated, and the credibility of the body of evidence is adequately evaluated. It is also important to present a comprehensive search strategy and assess the potential influence of publication bias on the study results. Additionally, reporting the funding sources of included studies is considered essential. The overall low quality of evidence is primarily due to significant risk of bias within the original studies and publication bias. Future RCTs should adopt more rigorous designs, increase sample sizes, and report outcomes using standardized methods to enhance the reliability of evidence syntheses.

### 4.2 rTMS interventions demonstrated a positive impact on upper limb motor function

In the published studies, all rTMS interventions demonstrated a positive impact on upper limb motor function. However, when the Action Research Arm Test (ARAT) was used as the measurement tool, the benefits of rTMS were not statistically significant (26). Compared to the ARAT, the Wolf Motor Function Test (WMFT) incorporates a greater number of complex movements related to daily activities. Since rTMS primarily influences isolated limb movements but has a limited effect on motor functions associated with activities of daily living (ADL), its impact on WMFT scores was less pronounced across studies.

Jiang’s study from 2024 showed high ratings in report completeness (96.3%), methodological quality (high), and quality of evidence for outcome measures (hand flexibility - high; upper limb functional activity - high; FMA-UE score - high; spasticity - moderate) (23). The results indicated that TMS could enhance the therapeutic effects of upper limb rehabilitation training after stroke. Changes in MAS scores and FMA-UE scores following TMS treatment were statistically significant. Although changes in ARAT scores were also statistically significant, the improvements were not substantial. Changes in hand NHPT scores and FMA-LE scores were not statistically significant. Another study also showed that TMS could enhance the therapeutic effects of post-stroke upper limb rehabilitation training (42).

The study by Al Jaber et al. (47) showed no significant improvement in the BBT following rTMS intervention. In alignment with this, Alhalabi H et al. (48) reported inconsistent ARAT outcomes across different phases of rehabilitation, indicating challenges in fine motor recovery with rTMS, which is consistent with the present findings. Lee et al. (49) demonstrated that rTMS significantly improved upper limb motor function and reduced spasticity, although the certainty of evidence was rated as low. Shen Y et al. (50) reported that rTMS led to significant improvements in both upper limb motor function and activities of daily living. These studies suggest that rTMS is effective in improving upper limb motor function in stroke patients, though its efficacy in fine motor recovery remains limited. The majority of studies support the positive effect of rTMS on enhancing ADL, which is consistent with the results of the present study.

The study by Zhang from 2017b indicated that both the resting motor threshold (rMT) and motor-evoked potentials (MEP) showed significant positive changes (44). Similarly, Zhao et al. (51) found that iTBS not only improved patients’ ADL but also shortened central motor conduction time. Chen K et al. (52)also reported that iTBS significantly reduced MEP latency. These findings suggest that neurophysiological indicators, such as cortical excitability, can serve as valuable outcome measures for assessing motor function recovery in stroke patients.

### 4.3 Adverse reactions

While TMS therapy has therapeutic effects, it also presents minor adverse reactions. These include mild headaches (14, 15, 19, 21, 25, 28, 30, 32, 37, 39, 43), a pricking sensation in the head (32, 34, 43), numbness in facial muscles (32), slight neck pain (14, 39), local discomfort at the site of stimulation (19), muscle spasms (23, 25, 28), muscle pain and fatigue (28), fatigue (21, 39), increased anxiety (14, 19, 21, 25, 30, 39, 43), and mild sleep disturbances (14, 19, 43). Notably, a severe adverse event was reported in only one instance; TMS was contraindicated for the patient after an electroencephalogram revealed epileptiform brain activity (32).

### 4.4 Feasibility suggestions for future clinical research directions

Current research predominantly focuses on upper limb recovery post-stroke, while studies applying TMS to lower limb rehabilitation remain limited (8). Emerging evidence indicates that rTMS targeting the lower limbs is a promising therapeutic approach, capable of improving FMA-LE scores in stroke patients. Furthermore, it generally promotes balance recovery and demonstrates advantages in enhancing gait speed, particularly during the acute and subacute stages (53). Recent investigations over the past two years reveal that cerebellar rTMS can significantly improve lower limb motor function. Zhu et al. (54) demonstrated that cerebellar rTMS significantly improved BBS scores, increased FMA-LE scores, and reduced Timed Up and Go (TUG) test and 10-Meter Walk Test (10MWT) durations. Three additional recent studies support cerebellar rTMS as a promising intervention for enhancing balance and lower limb motor function in stroke patients (41, 55, 56).

The relative scarcity of lower limb rTMS studies is partly attributable to the anatomical depth of the leg motor area within the interhemispheric fissure, which poses challenges for effective stimulation using standard figure-eight coils. iTBS stimulation of the cerebellum has been shown to improve visuomotor integration and enhance upper limb function in stroke patients (57), with only a few studies investigating its effects on lower limb muscles (27).

Cerebellar rTMS is an effective and safe technique that can improve lower limb motor function, balance, and ADL in stroke patients (37, 41). For future research, cerebellar rTMS holds significant promise as a therapeutic intervention to enhance balance and lower limb motor function in stroke patients. It is clinically practical and circumvents the limitation of insufficient stimulation depth associated with direct cortical leg area targeting (41).

Somatosensory impairment is known to hinder upper limb motor recovery, suggesting that residual sensorimotor control capacity may be a crucial factor in neurorehabilitation. Most published rTMS studies on motor recovery in stroke patients rarely report on sensorimotor deficits. Moreover, they frequently exclude patients with neuropsychiatric comorbidities such as aphasia, spatial neglect, or visual field defects, which are positively correlated with the severity of somatosensory impairments (58). In future, research should place greater emphasis on the relationship between sensory and motor recovery, integrating combined approaches for sensorimotor rehabilitation.

This study has its limitations: ① The number of original studies included in some research is limited, or the sample size of the original studies is small, resulting in a low combined effect value or high heterogeneity, which may affect the reliability of the results. ② The quality assessment of the included literature is subjective and largely based on scale ratings, introducing potential bias and constraining objectivity. ③ The search was restricted to published English literature, which may lead to publication bias.④ Intervention protocols across the original studies varied considerably and were not analyzed separately by category. ⑤ This study provides a general synthesis of the included data without delving into comparative effectiveness among different TMS protocols, precluding definitive conclusions regarding optimal stimulation parameters.

## 5 Summary

Systematic reviews and meta-analyses demonstrated that rTMS can ameliorate motor dysfunction in stroke patients. However, most of the existing RCTs are of low quality, and several issues persist: inadequate completeness and standardization of reported information, poor methodological quality of the included literature, and low quality of evidence for efficacy evaluation metrics.

Additionally, fundamental questions regarding optimal rTMS parameters—such as stimulation type, frequency, site, and the number of sessions required to achieve maximal efficacy—remain unanswered and warrant further investigation. Future efforts should prioritize high-quality, large-sample, multicenter clinical studies to validate the practical therapeutic benefits of rTMS in stroke rehabilitation.
