# Supplementary material for: The effectiveness of transcranial magnetic stimulation in ameliorating limb motor function disorders after stroke: an umbrella review
Source: Front Neurol. 2026 Mar 23;17:1741500. doi: 10.3389/fneur.2026.1741500 (PMC13051504; doi:10.3389/fneur.2026.1741500)
Supplement: Supplementary file 1 [file Table_1.docx]

**Table 1Specific characteristics tables of the literature were included**

| **Included in the literature** | **Number of studies (sample size)** | **Publication time of the original study/year** | **Experimental group** | **Control group** | **Stimulation site** | **Outcome indicators** | **Standard tools for methodological quality evaluation** | **For the GRADE evaluation** | **Main conclusion** |
| --- | --- | --- | --- | --- | --- | --- | --- | --- | --- |
| Chen 2022a | 45(2064) | Build the library-2022.02 | LF-rTMS、HF-rTMS、iTBS、cTBS、LF-rTMS+HF-rTMS、iTBS+cTBS | SM | affected, healthy, bilateral | ①④ | PEDro scale | N | For poststroke upper limb and hand dysfunction, both TBS and rTMS were significantly effective in the acute phase of stroke, and TBS was more effective than rTMS. However, in the subacute and chronic phases of stroke, rTMS was found to be more effective than TBS stimulation. |
| Chen 2022b | 25（535） | Build the library-2022.09 | LF-rTMS、HF-rTMS、iTBS、cTBS、iTBS+cTBS | SM | affected, healthy, bilateral, contralateral cerebellum | ①③⑤⑦⑧⑨⑩⑭ | PEDro scale | Y | The rTMS significantly improved upper limb function, hand function, muscle tone, balance and walking function, and ADL in chronic stroke patients, but not mobility or strength in the lower limbs. |
| Chen S 2023 | 10（236） | Build the library-2023.02 | iTBS | SM | The affected side M1, cerebellum, target muscle | ③④⑦⑪⑭ | Cochrane Risk of Bias assessment tool | N | Our results indicate that iTBS significantly improves motor impairment, functional activity and muscle tone in the upper limbs, thereby enhancing the ability of stroke patients to perform ADL, while no significant difference was found in MEP. |

| Gao 2022 | 13（334） | Build the library-2022.05 | iTBS+CT | SM+CT | affected | ③④⑤⑥⑦⑧⑩ | Cochrane Risk of Bias assessment tool、PEDro scale | N | The iTBS has good efficacy in improving the motor function in stroke patients. |
| --- | --- | --- | --- | --- | --- | --- | --- | --- | --- |
| Ghayour-Najafabadi 2019 | 15(385) | Build the library-2018.10 | LF-rTMS、HF-rTMS | SM | affected, healthy, cerebellum | ⑤⑧ | Cochrane Risk of Bias assessment tool | Y | rTMS has a positive effect on upper limb FMA mobility, balance ability and long-term prognosis. However, the data suggest insufficient evidence on the effectiveness of rTMS in improving lower limb function. |
| Graef 2016 | 8(199) | Build the library-2015.11 | rTMS+CT | SM+CT | affected, healthy | ①③④⑦⑭ | Cochrane Risk of Bias assessment tool | N | The current literature is insufficient to support the hypothesis that combining rTMS with upper limb training has a greater impact on upper limb function than upper limb training alone. |
| Hao 2013 | 19(588) | Build the library-2012.04 | LF-rTMS、HF-rTMS、LF-rTMS+HF-rTMS | SM | affected, healthy, bilateral | ③⑩⑭ | Cochrane Risk of Bias assessment tool | N | Routine use of rTMS is not recommended in stroke patients until validation of the efficacy of rTMS in high-quality, large-scale randomized controlled trials. |
| He 2020 | 28(888) | Build the library-2018.09 | LF-rTMS、HF-rTMS、LF-rTMS+HF-rTMS、rTMS+CT、LF-rTMS/HF-rTMS+CT+Medicine | SM、SM+CT、SM+CT+ Medicine | affected, healthy | ①②④⑤⑥⑩⑬ | Cochrane Risk of Bias assessment tool | Y | Advocate the use of rTMS to promote motor recovery in stroke patients low frequency rTMS has a positive effect on grip strength and lower limb function assessed by FMA. |

| Hsu 2012 | 18（392） | Build the library-2011.01 | LF-rTMS、HF-rTMS、iTBS、cTBS | SM | affected, healthy, bilateral | ③⑫⑭ | The revised quality screening table presented by Moher et al | N | The rTMS has clinically positive effects on motor recovery of the affected upper limbs in stroke patients. |
| --- | --- | --- | --- | --- | --- | --- | --- | --- | --- |
| Huang 2022 | 18（401） | Build the library-2021.11 | iTBS+CT+Medicine | SM+CT+Medicine | affected | ④⑩⑪⑫ | Cochrane Risk of Bias assessment tool | N | Identified the efficacy of iTBS in improving motor cortical plasticity, motor function, and daily function in stroke patients. |
| Jiang 2024 | 16(438) | Build the library-2023.08 | cTBS、iTBS、cTBS+iTBS | SM | affected, healthy, bilateral, cerebellum | ①③④⑤⑦⑧⑭ | Cochrane Risk of Bias assessment tool | Y | Significant for spasticity, significant improvement in lower limb FMA, and motor function results suggest that targeting cerebral cortex M1 with TBS when assessing fine motor and hand skills using NHPT was ineffective, did not find any benefit of TBS on recovery of lower limb motor function after stroke. |
| Kang 2020 | 9 （263） | Build the library-2019.04 | LF-rTMS/HF-rTMS+CT | SM+CT | affected, healthy, cerebellum | ⑧ | PEDro scale | N | Short-term therapeutic effects on post-stroke functional balance and postural control were significantly positive. |
| Le 2014 | 8（273） | 1990-2012.01 | LF-rTMS、HF-rTMS | SM | affected, healthy | ①⑪⑫⑭ | PEDro scale | N | rTMS improves hand motor function in stroke patients. |

| Li 2024 | 19(865) | 2014.03.28-2023.03.28 | LF-rTMS/HF-rTMS+CT、LF-rTMS+HF-rTMS+CT | SM+CT | affected, healthy, bilateral | ③④⑩⑬ | Cochrane Risk of Bias assessment tool | N | The rTMS improved the lower limb FMA, Barthel index, and Brunnstrom recovery phase. |
| --- | --- | --- | --- | --- | --- | --- | --- | --- | --- |
| Li 2018 | 9(220) | 2012-2017 | LF-rTMS、HF-rTMS、LF-rTMS/HF-rTMS+CT | SM、SM+CT | affected, healthy | ⑤⑧⑨⑪ | PEDro scale | N | Ipsilateral stimulation of rTMS, especially HF-rTMS, had significant effects on increasing walking speed. |
| Liu 2021 | 7(554) | Build the library-2021.05 | LF-rTMS/HF-rTMS+CT、LF-rTMS+HF-rTMS+CT | SM+CT | affected, healthy, bilateral | ⑤⑦⑧⑩⑭ | Cochrane Risk of Bias assessment tool、PEDro scale | N | The rTMS is effective in improving the spasticity and activities of daily living. LF-rTMS has a positive clinical effect on enhancing motor function in patients with lower limb spasticity after stroke. |
| McIntyre 2018 | 10（273) | 1980.01-2015.04 | LF-rTMS+CT、LF-rTMS、LF-rTMS+HF-rTMS+CT | SM、SM+CT | healthy, bilateral | ⑦  ⑭ | PEDro scale | N | The available evidence for the use of rTMS to improve spasms after stroke is limited. |
| Narayan 2022 | 2(55) | 2001-2019 | LF-rTMS+CT | SM+CT | affected | ①⑭ | NR | N | Patients who received the TMS intervention showed improvement in motor scale function compared to the sham group. |
| Ni 2021 | 8（242） | Build the library-2021.03 | LF-rTMS、HF-rTMS | SM | affected, healthy | ⑤⑧⑨ | NR | N | The rTMS improved lower limb function in stroke patients. |

| Tang 2022 | 15（449） | Build the library-2021.12.31 | HF-rTMS/iTBS+CT | SM+CT | affected | ①②④⑪⑭ | Cochrane Risk of Bias assessment tool、PEDro scale | N | Excitatory rTMS significantly improved upper limb motor function, but not in patients lasting longer than 3 months. |
| --- | --- | --- | --- | --- | --- | --- | --- | --- | --- |
| Tian 2011 | 2(72) | 1989.01-2010.12 | TMS+CT | CT | motor cortex | ⑬ | Cochrane Risk of Bias assessment tool | N | TMS had no obvious effect on improving the NIHSS score of neurological function after stroke. |
| Tung 2019 | 8（169） | Build the library-2019.01.25 | LF-rTMS/HF-rTMS+CT | SM+CT | Left dorsolateral prefrontal cortex of the affected side / healthy side | ⑤⑨⑪⑭ | PEDro scale | N | The rTMS significantly improved the physical function in the lower limbs. |
| van Lieshout 2019 | 38（1074） | Build the library-2018.02 | LF-rTMS、HF-rTMS、iTBS、cTBS、LF-rTMS+HF-rTMS+CT、iTBS+cTBS+CT | SM+CT | affected, healthy, bilateral | ①③④ | PEDro scale | N | According to the FMA, rTMS appears to be more beneficial only when treatment is initiated in the first month after stroke. A functional level test may be more sensitive to detecting beneficial effects of rTMS on upper limb function than an activity level test. |
| Vaz 2019 | 3(92) | 2012-2015 | LF-rTMS/HF-rTMS+CT | SM+CT | affected, healthy | ⑨ | Cochrane Risk of Bias assessment tool | Y | TMS combined with other therapies positively influenced TMS compared with sham PTMS |

| Veldema 2022 | 9(212) | Build the library-2021.03.31 | LF-rTMS、HF-rTMS、iTBS | SM | affected, healthy, bilateral | ⑨ | PEDro scale | N | Can effectively improve gait, balance and lower limb motor function in stroke survivors. |
| --- | --- | --- | --- | --- | --- | --- | --- | --- | --- |
| Wang 2024 | 13(542) | Build the library-2023.10.20 | LF-rTMS/HF-rTMS/iTBS/cTBS+CT | SM+CT | The affected side / healthy side cerebellum | ⑧⑩⑭ | PEDro scale | N | Cerebellar TMS may still improve post-intervention BBS and post-intervention ADL. |
| Wang 2022 | 11(267) | Build the library-2021.12 | HF-rTMS/LF-rTMS+CT、iTBS、cTBS | SM、SM+CT | affected, healthy | ⑦ | Cochrane Risk of Bias assessment tool | N | The rTMS had a significant effect on improving the spasticity. |
| Xiang 2019 | 42（1168） | 2005.01-2018.12 | LF-rTMS、HF-rTMS、TBS | SM+CT | healthy, affected | ⑥⑩⑭ | PEDro scale | N | The rTMS had positive effects on limb motor recovery and activities of daily living and altered cortical excitability. |
| Xu 2021 | 5(126) | Build the library-2020.02 | LF-rTMS/HF-rTMS/iTBS+CT、LF-rTMS+HF-rTMS | SM+CT | bilateral, affected, healthy | ⑦ | Cochrane Risk of Bias assessment tool | N | Compared with sham treatment, rTMS has no significant benefit on MAS in patients with poststroke spasticity. |
| Zeng 2024 | 10（420） | Build the library-2023.05.01 | LF-rTMS/HF-rTMS/iTBS+CT | SM+CT | The affected side / healthy side cerebellum | ⑤⑧ | Cochrane Risk of Bias assessment tool | N | Cerebellar TMS significantly improved balance ability and lower limb motor function in stroke patients. |
| Zhang 2024 | 29（779） | 2000.01.01-2000.08.29 | iTBS、cTBS、cTBS + iTBS、LF rTMS + iTBS | SM、LF-TMS+SM | affected, healthy, bilateral | ③④ | PEDro scale | N | TBS is an effective brain stimulation therapy that can enhance the therapeutic effect of upper limb rehabilitation training after stroke. |

| Zhang 2017a | 34（904） | Build the library-2016.10.30 | LF-rTMS、HF-rTMS、iTBS、cTBS | SM | affected, healthy | ①⑭ | The revised quality screening table presented by Moher et al | N | Five rTMS treatments can effectively and persistently improve the upper limb movement disorders caused by stroke. |
| --- | --- | --- | --- | --- | --- | --- | --- | --- | --- |
| Zhang 2017b | 22（619） | Build the library-2017.06.31 | LF-rTMS | SM+CT | affected | ①②③④⑪⑫ | The revised quality screening table presented by Moher et al | N | LF-rTMS significantly improved the functional recovery of the upper limbs, especially in the hands after stroke. |
| Zhang 2023 | 13(428) | Build the library-2022.07.11 | LF-rTMS/HF-rTMS+CT、LF-rTMS+HF-rTMS+CT | SM+CT | affected, healthy, bilateral, Cerebellum on the healthy side / affected side | ⑤⑧⑩⑪ | PEDro scale | Y | Low-frequency rTMS significantly improved BI scores, while high-frequency rTMS and iTBS did not significantly improve BI scores. |

Note: LF-rTMS: Low-frequency repetitive transcranial magnetic stimulation; HF-rTMS: High-frequency repetitive transcranial magnetic stimulation; rTMS: Repetitive transcranial magnetic stimulation; TBS: Theta burst stimulation; iTBS: Intermittent theta burst stimulation; cTBS: Continuous theta burst stimulation; CT: Conventional treatment; Medicine: Pharmacotherapy; SM: Sham rTMS; ADL: Activities of daily living; MEP: Motor evoked potential; FMA: Fugl-Meyer Assessment; NHPT: Nine Hole Peg Test; NIHSS: National Institutes of Health Stroke Scale; BBS: Berg Balance Scale; BI: Barthel Index; ① Hand flexibility; ② Hand strength; ③ Upper limb functional activity; ④ Fugl-Meyer upper limb function score; ⑤ Lower limb motor function; ⑥ Limb motor function; ⑦ Spasticity; ⑧ Balance ability; ⑨ Walking ability; ⑩ Activities of daily living; ⑪ Motor evoked potential; ⑫ Motor threshold; ⑬ Severity of stroke; ⑭ Adverse reactions; Cochrane Risk of Bias assessment tool: Cochrane tool for assessing risk of bias; PEDro scale: Physiotherapy Evidence Database scale; N: No; Y: Yes.
