# Supplementary material for: The effectiveness of transcranial magnetic stimulation in ameliorating limb motor function disorders after stroke: an umbrella review
Source: Front Neurol. 2026 Mar 23;17:1741500. doi: 10.3389/fneur.2026.1741500 (PMC13051504; doi:10.3389/fneur.2026.1741500)
Supplement: Supplementary file 3 [file Table_3.docx]

**Table 3AMSTAR2 Methodological quality evaluation**

| **Included in the study** | **Q1** | **Q2*** | **Q3** | **Q4*** | **Q5** | **Q6** | **Q7*** | **Q8** | **Q9*** | **Q10** | **Q11*** | **Q12** | **Q13*** | **Q14** | **Q15*** | **Q16** | **Quality evaluation grade (high, medium, low, very low)** |
| --- | --- | --- | --- | --- | --- | --- | --- | --- | --- | --- | --- | --- | --- | --- | --- | --- | --- |
| Chen 2022a | Y | Y | Y | Y | N | Y | Y | Y | Y | N | Y | Y | Y | Y | N | Y | low |
| Chen 2022b | Y | Y | Y | Y | N | Y | Y | Y | Y | N | Y | Y | N | Y | N | Y | very low |
| Chen S 2023 | Y | Y | Y | Y | Y | Y | Y | Y | Y | N | Y | Y | N | Y | N | Y | very low |
| Gao 2022 | Y | N | Y | Y | Y | Y | Y | Y | Y | N | Y | Y | N | Y | N | Y | very low |
| Ghayour-Najafabadi 2019 | Y | Y | Y | Y | Y | Y | Y | Y | Y | N | Y | Y | Y | N | N | N | low |
| Graef 2016 | Y | N | Y | Y | Y | Y | Y | Y | Y | N | Y | Y | Y | Y | N | N | very low |
| Hao 2013 | Y | N | Y | Y | Y | Y | N | Y | Y | N | Y | Y | Y | Y | Y | PY | very low |
| He 2020 | Y | Y | Y | Y | N | Y | Y | Y | Y | N | Y | Y | Y | Y | Y | N | medium |
| Hsu 2012 | Y | N | Y | Y | Y | Y | Y | Y | Y | N | Y | Y | N | Y | N | PY | very low |
| Huang 2022 | Y | Y | Y | Y | Y | Y | Y | Y | Y | N | Y | Y | Y | Y | Y | Y | high |
| Jiang 2024 | Y | Y | Y | Y | Y | Y | Y | Y | Y | N | Y | Y | Y | Y | Y | Y | high |
| Kang 2020 | Y | N | Y | Y | Y | Y | Y | Y | Y | N | Y | Y | N | Y | N | Y | very low |
| Le 2014 | Y | N | Y | Y | Y | Y | N | Y | Y | N | Y | Y | N | N | N | Y | very low |
| Li 2024 | Y | Y | Y | Y | Y | Y | Y | Y | Y | N | Y | Y | N | N | N | Y | very low |
| Li 2018 | Y | N | Y | Y | Y | Y | Y | Y | Y | N | Y | Y | Y | Y | N | PY | very low |
| Liu 2021 | Y | N | Y | Y | Y | Y | Y | Y | Y | N | Y | Y | N | Y | N | Y | very low |
| McIntyre 2018 | Y | N | Y | Y | N | N | Y | Y | Y | N | Y | Y | N | N | N | N | very low |
| Narayan 2022 | Y | Y | Y | Y | N | N | Y | Y | N | N | Y | N | N | N | N | Y | very low |
| Ni 2021 | Y | N | Y | Y | N | N | Y | Y | N | N | Y | N | N | Y | Y | Y | very low |
| Tang 2022 | Y | Y | Y | Y | Y | Y | Y | Y | Y | N | Y | Y | Y | Y | N | Y | low |
| Tian 2011 | Y | N | Y | Y | N | Y | Y | Y | Y | Y | Y | Y | Y | N | Y | PY | low |
| Tung 2019 | Y | N | Y | Y | Y | Y | Y | Y | Y | N | Y | Y | Y | Y | N | Y | very low |
| van Lieshout 2019 | Y | N | Y | Y | N | N | Y | Y | Y | N | Y | Y | Y | Y | Y | Y | low |
| Vaz 2019 | Y | N | Y | Y | Y | Y | Y | Y | Y | N | Y | Y | N | Y | N | Y | very low |
| Veldema 2022 | Y | N | Y | Y | Y | N | N | Y | Y | N | Y | Y | N | N | N | Y | very low |
| Wang 2024 | Y | N | Y | Y | Y | Y | Y | Y | Y | N | Y | Y | Y | Y | N | Y | very low |
| Wang 2022 | Y | N | Y | Y | Y | N | N | Y | Y | N | Y | Y | N | Y | N | Y | very low |
| Xiang 2019 | Y | N | Y | Y | Y | Y | Y | Y | Y | N | Y | Y | Y | Y | Y | Y | low |
| Xu 2021 | Y | N | Y | Y | Y | N | N | Y | Y | N | Y | Y | Y | Y | N | Y | very low |
| Zeng 2024 | Y | Y | Y | Y | Y | Y | Y | Y | Y | N | Y | Y | N | N | N | Y | very low |
| Zhang 2024 | Y | Y | Y | Y | Y | Y | Y | Y | N | N | Y | N | N | N | N | Y | very low |
| Zhang 2017a | Y | N | Y | Y | N | Y | N | Y | Y | N | Y | Y | Y | N | Y | Y | very low |
| Zhang 2017b | Y | Y | Y | Y | Y | Y | N | Y | Y | N | Y | Y | N | N | N | Y | very low |
| Zhang 2023 | Y | Y | Y | Y | N | Y | Y | Y | Y | N | Y | N | N | Y | N | Y | very low |

Note: Q1: including PICO elements Q2 *: registered study protocol Q3: explain the reasons for included study type Q4 *: comprehensive search literature Q5: two people independently screening literature Q6: two people independently extracted data Q7 *: provide study list and reasons for excluding studies Q8: detailed description of included study basic characteristics Q9 *: Use reasonable tools to assess the risk of bias Q10: report included study funding source Q11 *: using appropriate methods results Q12: consider the potential impact of bias risk Q13 *: discuss the impact of bias risk on study results Q14: explain the heterogeneity in the study Q1 5 *: Investigate the impact of publication bias on findings Q16: Reporting conflicts of interest and funding.* For important entries, Y: Yes N: No PY: Partial Yes.
