# Supplementary material for: The effectiveness of transcranial magnetic stimulation in ameliorating limb motor function disorders after stroke: an umbrella review
Source: Front Neurol. 2026 Mar 23;17:1741500. doi: 10.3389/fneur.2026.1741500 (PMC13051504; doi:10.3389/fneur.2026.1741500)
Supplement: Supplementary file 4 [file Table_4.docx]

**Table 4GRADE quality of evidence**

| **Outcome measures and the included studies** | **Number of studies on outcome measures** | **Risk of bias** | **Inconsistency** | **Indirect** | **Uncertainty** | **Publication bias** | **Evidence grading** | **Effect quantity** | **95%CI** | **I2** |
| --- | --- | --- | --- | --- | --- | --- | --- | --- | --- | --- |
| ①Hand flexibility |  |  |  |  |  |  |  |  |  |  |
| Chen 2022a | 8 | 0 | 0 | 0 | 0 | -1d | middle | SMD=0.38 | [0.19, 0.58] | 0.00% |
| Chen 2022b | 6 | 0 | 0 | 0 | 0 | -1d | middle | SMD=0.45 | [0.08, 0.83] | 0.00% |
| Graef 2016 | 2 | -1a | 0 | 0 | -1c | -1d | very low | MD=2.87 | [−3.59, 9.33] | 0.00% |
| Tang 2022 | 6 | 0 | 0 | 0 | 0 | -1d | middle | SMD=0.76 | [0.39, 1.14] | 47.00% |
| He 2020 | 3 | -1a | 0 | 0 | -1c | 0 | low | SMD=-0.246 | [-0.84, 0.35] | 39.50% |
| Jiang 2024 | 4 | 0 | 0 | 0 | 0 | 0 | high | SMD=-0.01 | [-0.44, 0.43] | 40.00% |
| Le 2014 | 4 | -1a | 0 | 0 | 0 | 0 | middle | SMD=0.580 | [0.12, 1.04] | 0.00% |
| Le 2014（Hand function） | 3 | -1a | 0 | 0 | 0 | 0 | middle | SMD=-0.82 | [-1.30, -0.33] | 0.00% |
| Narayan 2022 | 2 | -1a | 0 | 0 | 0 | -1d | low | MD=0.75 | [0.03, 1.48] | 0.00% |
| van Lieshout 2019 | 28 | -1a | -1b | 0 | 0 | -1d | very low | SMD=0.43 | [0.21, 0.65] | 60.00% |
| Zhang 2017a（short-term） | 27 | -1a | 0 | 0 | 0 | 0 | middle | SMD=0.43 | [0.30, 0.56] | 0.00% |
| Zhang 2017a（long-term） | 11 | -1a | 0 | 0 | 0 | 0 | middle | SMD=0.49 | [0.29, 0.68] | 38.70% |
| Zhang 2017b | 6 | -1a | 0 | 0 | 0 | 0 | middle | SMD=0.75 | [0.44, 1.06] | 0.00% |
| ②Hand strength |  |  |  |  |  |  |  |  |  |  |
| He 2020 | 5 | 0 | 0 | 0 | -1c | 0 | middle | SMD=1.147 | [0.76, 1.53] | 23.40% |
| Tang 2022 | 5 | 0 | -1b | 0 | 0 | -1d | low | SMD=0.53 | [0.04, 1.01] | 56.00% |
| Zhang 2017b | 11 | -1a | 0 | 0 | 0 | 0 | middle | SMD=0.49 | [0.22, 0.76] | 12.00% |
| ③Upper limb functional activity |  |  |  |  |  |  |  |  |  |  |
| van Lieshout 2019 | 20 | -1a | 0 | 0 | 0 | -1d | low | SMD=0.17 | [-0.19, 0.44] | 49.00% |
| Zhang 2024 | 14 | -1a | 0 | 0 | 0 | -1d | low | Hedges'g=0.500 | [0.27, 0.73] | 34.12 % |

| Gao 2022 | 2 | 0 | -2b | 0 | -1c | -1d | very low | SMD=2.11 | [-0.59, 4.80] | 94.00% |
| --- | --- | --- | --- | --- | --- | --- | --- | --- | --- | --- |
| Graef 2016 | 4 | -1a | -1b | 0 | 0 | -1d | very low | MD=0.73 | [-0.93, 2.39] | 62.00% |
| Graef 2016 | 3 | -1a | -1b | 0 | 0 | -1d | very low | MD=3.60 | [-3.73, 10.94] | 53.00% |
| Chen S 2023 | 5 | -1a | 0 | 0 | -1c | -1d | very low | WMD=3.72 | [2.13, 5.30] | 0.00% |
| Gao 2022 | 5 | 0 | -2b | 0 | -1c | -1d | very low | SMD=2.28 | [0.83, 3.73] | 87.00% |
| Graef 2016 | 3 | -1a | 0 | 0 | -1c | -1d | very low | MD=−0.32 | [−6.26, 5.62] | 0.00% |
| Jiang 2024 | 5 | 0 | 0 | 0 | 0 | 0 | high | MD=3.35 | [2.78, 3.91] | 36.00% |
| Li 2024 | 5 | -1a | -2b | 0 | 0 | -1d | very low | SMD=0.25 | [−0.35, 0.84] | 79.00% |
| Chen 2022b | 15 | 0 | -1b | 0 | 0 | -1d | low | SMD=0.49 | [0.08, 0.89] | 63.90% |
| Hao 2013 | 4 | 0 | -2b | 0 | 0 | -1d | very low | SMD=0.51 | [-0.99, 2.01] | 87.60% |
| Hsu 2012 | 17 | -1a | 0 | 0 | 0 | -1d | low | SMD=0.55 | [0.37, 0.72] | 0.00% |
| Zhang 2017b | 10 | -1a | 0 | 0 | 0 | 0 | middle | SMD=0.32 | [0.09, 0.55] | 0.00% |
| ④The Fugl-Meyer upper limb score |  |  |  |  |  |  |  |  |  |  |
| Chen 2022a | 39 | 0 | 0 | 0 | 0 | -1d | middle | SMD=0.91 | [0.61, 1.22] | 0.00% |
| Chen S 2023 | 6 | -1a | 0 | 0 | 0 | -1d | low | WMD=3.20 | [1.42, 4.97] | 28.00% |
| Gao 2022 | 6 | 0 | -1b | 0 | 0 | -1d | low | SMD=0.97 | [0.07, 1.97] | NR |
| Graef 2016 | 4 | -1a | 0 | 0 | 0 | -1d | low | MD=0.50 | [−2.20, 3.20] | 0.00% |
| He 2020 | 15 | 0 | -1b | 0 | -1c | 0 | low | SMD=0.69 | [0.41, 0.98] | 55.80% |
| Huang 2022 | 8 | -1a | 0 | 0 | 0 | -1d | low | MD=2.70 | [-0.02, 5.42] | 13.00% |
| Jiang 2024 | 8 | 0 | 0 | 0 | 0 | 0 | high | MD=3.89 | [0.60, 7.18] | 88.00% |
| Li 2024 | 14 | -1a | 0 | 0 | 0 | -1d | low | MD=1.87 | [0.88, 2.86] | 41.00% |
| Tang 2022 | 11 | 0 | -1b | 0 | 0 | -1d | low | MD=5.88 | [3.32, 8.43] | 58.00% |
| van Lieshout 2019 | 15 | -1a | -1b | 0 | 0 | -1d | very low | MD=4.25 | [1.39, 7.11] | 71.00% |
| Zhang 2017b | 7 | -1a | -1b | 0 | 0 | 0 | low | SMD=0.29 | [−0.06, 0.64] | 52.00% |
| Zhang 2024 | 12 | -1a | -2b | 0 | 0 | -1d | very low | Hedges'g=0.646 | [0.21, 1.08] | 76.15% |

| ⑤Lower limb motor function |  |  |  |  |  |  |  |  |  |  |
| --- | --- | --- | --- | --- | --- | --- | --- | --- | --- | --- |
| Chen 2022b | 3 | 0 | 0 | 0 | -1c | -1d | low | SMD=0.15 | [-0.37, 0.67] | 0.00% |
| Gao 2022 | 4 | 0 | -1b | 0 | -1c | -1d | very low | SMD=1.03 | [0.00, 2.06] |  |
| Ghayour-Najafabadi 2019 | 6 | 0 | 0 | 0 | -1c | -1d | low | SMD=0.01 | [-0.29, 0.31] | 0.00% |
| He 2020 | 6 | 0 | 0 | 0 | -1c | 0 | middle | SMD=0.54 | [0.26, 0.82] | 0.00% |
| Jiang 2024 | 4 | -1a | 0 | 0 | 0 | 0 | middle | MD=0.44 | [-0.76, 1.64] | 2.00% |
| Li 2018 | 3 | -1a | 0 | 0 | -1c | 0 | low | MD=0.50 | [-0.68, 1.68] | 0.00% |
| Liu 2021 | 7 | -1a | -2b | 0 | 0 | -1d | very low | SMD=0.54 | [0.37, 0.72] | 84.00% |
| Tung 2019 | 4 | 0 | 0 | 0 | -1c | -1d | low | SMD=0.63 | [0.16, 1.10] | 1.00% |
| Zeng 2024 | 4 | 0 | 0 | 0 | 0 | -1d | middle | MD=2.11 | [1.45, 2.78] | 3.00% |
| Zhang 2023 | 9 | -1a | 0 | 0 | 0 | -1d | low | MD=−0.73 | [−1.80, 0.34] | 20.00% |
| Ni 2021 | 8 | -1a | -2b | 0 | 0 | -1d | very low | SMD=1.65 | [0.53, 2.76] | 90.30% |
| ⑥Body motor function |  |  |  |  |  |  |  |  |  |  |
| Gao 2022 | 10 | 0 | -2b | 0 | 0 | -1d | very low | SMD=0.99 | [0.35, 1.62] | 80.00% |
| He 2020 | 21 | 0 | 0 | 0 | -1c | 0 | middle | SMD=0.635 | [0.42, 0.85] | 44.90% |
| Xiang 2019 | 42 | -1a | 0 | 0 | 0 | 0 | middle | SMD=0.50 | [0.39, 0.60] | 0.00% |
| ⑦convulsion |  |  |  |  |  |  |  |  |  |  |
| Chen 2022b | 7 | 0 | 0 | 0 | 0 | -1d | middle | WMD=-0.37 | [-0.51, -0.24] | 0.00% |
| Chen S 2023 | 5 | -1a | 0 | 0 | -1c | -1d | very low | WMD=-0.56 | [-0.85, -0.28] | 31.00% |
| Gao 2022 | 4 | 0 | -2b | 0 | 0 | -1d | very low | SMD=-0.71 | [-1.88, 0.47] | 85.00% |
| Graef 2016 | 2 | -1a | 0 | 0 | -1c | -1d | very low | MD=−0.31 | [−0.78, 0.17] | 43.00% |
| Jiang 2024 | 5 | -1a | 0 | 0 | 0 | 0 | middle | MD=-0.44 | [-0.77, 0.12] | 0.00% |
| Liu 2021 | 5 | -1a | 0 | 0 | 0 | -1d | low | SMD=-0.24 | [-0.45, -0.03] | 0.00% |
| McIntyre 2018 | 6 | -1a | -1b | 0 | 0 | -1d | very low | SMD=0.609 | [0.29, 0.93] | 55.40% |
| Wang 2022 | 11 | -1a | 0 | 0 | 0 | -1d | low | MD=-0.40 | [-0.56, -0.25] | 3.00% |
| Xu 2021 | 5 | -1a | 0 | 0 | -1c | -1d | very low | WMD=−0.29 | [−0.58, 0.00] | 0.00% |

| ⑧ balanced capacity |  |  |  |  |  |  |  |  |  |  |
| --- | --- | --- | --- | --- | --- | --- | --- | --- | --- | --- |
| Chen 2022b | 3 | 0 | 0 | 0 | -1c | -1d | low | SMD=0.95 | [0.43, 1.46] | 48.50% |
| Ghayour-Najafabadi 2019 | 3 | 0 | -1b | 0 | -1c | 0 | low | SMD=-0.67 | [-1.08, -0.26] | 72.00% |
| Liu 2021 | 2 | -1a | 0 | 0 | -1c | -1d | very low | SMD=-0.01 | [-0.33, 0.30] | 0.00% |
| Wang 2024 | 4 | 0 | 0 | 0 | 0 | -1d | middle | MD=-1.51 | [-2.8, -0.22] | 0.00% |
| Gao 2022 | 2 | 0 | 0 | 0 | -1c | -1d | low | SMD=0.32 | [-0.24, 0.88] | 0.00% |
| Jiang 2024 | 3 | -1a | -1b | 0 | 0 | 0 | low | MD=2.61 | [ -0.74, 5.95] | 73.00% |
| Wang 2024 | 8 | 0 | -1b | 0 | 0 | -1d | low | MD=4.24 | [2.19, 6.29] | 74.00% |
| Zeng 2024 | 8 | 0 | -2b | 0 | 0 | -1d | very low | MD=4.54 | [2.33, 6.74] | 78.00% |
| Zhang 2023 | 4 | -1a | -1b | 0 | -1c | -1d | very low | MD=−0.40 | [−4.81, 4.01] | 69.00% |
| Kang 2020 | 9 | -1a | 0 | 0 | 0 | -1d | low | SMD=0.475 | [0.19, 0.76] | 36.60% |
| Li 2018 | 3 | -1a | 0 | 0 | -1c | -1d | very low | SMD=0.10 | [-0.26, 0.45] | 0.00% |
| Ghayour-Najafabadi 2019 | 5 | 0 | -1b | 0 | -1c | 0 | low | SMD=0.38 | [0.07, 0.69] | 51.00% |
| Ni 2021 | 5 | -1a | -2b | 0 | -1c | -1d | very low | SMD=3.54 | [1.45, 5.63] | 95.40% |
| Wang 2024 | 3 | 0 | 0 | 0 | 0 | -1d | middle | MD=-3.25 | [-9.76, 3.27] | 0.00% |
| Wang 2024 | 2 | 0 | 0 | 0 | 0 | -1d | middle | MD=-4.47 | [-5.8, -3.14] | 0.00% |
| Wang 2024 | 3 | 0 | 0 | 0 | 0 | -1d | middle | MD=-4.37 | [-5.58, -3.15] | 0.00% |
| ⑨Walking ability |  |  |  |  |  |  |  |  |  |  |
| Chen 2022b | 6 | 0 | 0 | 0 | 0 | -1d | middle | SMD=0.36 | [0.01, 0.70] | 7.80% |
| Ni 2021 | 6 | -1a | -2b | 0 | 0 | -1d | very low | SMD=3.31 | [1.38, 5.24] | 92.10% |
| Tung 2019 | 3 | 0 | 0 | 0 | -1c | -1d | low | SMD=1.13 | [0.57, 1.70] | 0.00% |
| Vaz 2019 | 3 | -1a | 0 | 0 | 0 | -1d | low | MD=0.09 | [0.05, 0.13] | 0.00% |
| Li 2018 | 6 | -1a | 0 | 0 | 0 | -1d | low | SMD=0.64 | [0.32, 0.95] | 0.00% |
| Veldema 2022 | 9 | 0 | 0 | 0 | 0 | -1d | middle | SMD=0.34 | [-0.46, 1.15] | 0.00% |
| ⑩Ability of daily living activities |  |  |  |  |  |  |  |  |  |  |

| Chen 2022b | 9 | 0 | 0 | 0 | 0 | -1d | middle | SMD=0.41 | [0.15, 0.67] | 36.50% |
| --- | --- | --- | --- | --- | --- | --- | --- | --- | --- | --- |
| Huang 2022 | 3 | -1a | 0 | 0 | 0 | -1d | low | MD=7.34 | [0.47, 14.21] | 0.00% |
| Liu 2021 | 2 | -1a | 0 | 0 | -1c | -1d | very low | MD=6.14 | [3.93,8.35] | 40.00% |
| Gao 2022 | 3 | 0 | 0 | 0 | -1c | -1d | low | SMD=-0.04 | [-0.39, 0.48] | 3.00% |
| Hao 2013 | 2 | 0 | -2b | 0 | 0 | -1d | very low | MD=15.92 | [-2.11, 33.95] | 97.00% |
| Li 2024 | 6 | -1a | -1b | 0 | 0 | -1d | very low | MD=9.73 | [4.57, 14.89] | 58.00% |
| Zhang 2023 | 8 | -1a | -2b | 0 | 0 | -1d | very low | MD=5.87 | [0.99, 10.76] | 86.00% |
| Xiang 2019 | 7 | -1a | 0 | 0 | 0 | 0 | middle | SMD=0.82 | [0.59, 1.05] | 0.00% |
| He 2020 | 11 | -1a | 0 | 0 | -1c | 0 | low | SMD=0.58 | [0.38, 0.78] | 0.00% |
| Wang 2024 | 6 | 0 | -1b | 0 | 0 | -1d | low | MD=7.75 | [4.33, 11.17] | 56.00% |
| ⑪Motion Evoked Potential |  |  |  |  |  |  |  |  |  |  |
| Chen S 2023 (affected side) | 3 | -1a | 0 | 0 | -1c | -1d | very low | SMD=0.35 | [-0.21, 0.90] | 0.00% |
| Chen S 2023 (unaffected side) | 2 | -1a | 0 | 0 | -1c | -1d | very low | SMD=0.35 | [-0.18, 0.87] | 0.00% |
| Huang 2022 (affected side) | 1 | -1a | -2b | 0 | -1c | -1d | very low | MD=0.70 | [0.40, 1.00] |  |
| Huang 2022 (unaffected side) | 3 | -1a | -2b | 0 | 0 | -1d | very low | MD=1.63 | [1.43, 1.84] | 95.00% |
| Huang 2022 | 4 | -1a | -1b | 0 | 0 | -1d | very low | MD=1.34 | [1.17, 1.51] | 96.00% |
| Le 2014 (affected side) | 3 | -1a | -1b | 0 | -1c | 0 | very low | SMD=0.89 | [-0.55, 2.33] | 73.00% |
| Li 2018 (affected side) | 2 | -1a | -2b | 0 | -1c | -1d | very low | MD=0.21 | [-0.11, 0.54] | 90.00% |
| Li 2018 (unaffected side) | 1 | -1a | 0 | 0 | -1c | -1d | very low | MD=0.09 | [-0.16, -0.02] | 0.00% |
| Tang 2022 (affected side) | 3 | 0 | 0 | 0 | -1c | -1d | low | SMD=0.82 | [0.32, 1.33] | 0.00% |
| Tang 2022 (unaffected side) | 2 | 0 | 0 | 0 | -1c | -1d | low | SMD=0.22 | [-0.42, 0.86] | 0.00% |

| Tung 2019 | 3 | 0 | 0 | 0 | -1c | -1d | low | SMD=1.13 | [0.56, 1.70] | 0.00% |
| --- | --- | --- | --- | --- | --- | --- | --- | --- | --- | --- |
| Zhang 2017b (affected side) | 4 | -1a | 0 | 0 | 0 | 0 | middle | SMD=0.38 | [0.02, 0.74] | 0.00% |
| Zhang 2017b (unaffected side) | 8 | -1a | 0 | 0 | 0 | 0 | middle | SMD=-0.83 | [-1.13, -0.54] | 18.00% |
| Zhang 2023 (affected side) | 5 | -1a | -2b | 0 | -1c | -1d | very low | MD=−6.80 | [−53.15, 39.55] | 78.00% |
| ⑫Motion threshold |  |  |  |  |  |  |  |  |  |  |
| Hsu 2012 (affected side) | 5 | -1a | 0 | 0 | -1c | -1d | very low | SMD=0.30 | [-0.09, 0.68] | 0.00% |
| Le 2014 | 3 | -1a | 0 | 0 | 0 | 0 | high | SMD=-0.26 | [-0.82, 0.30] | 0.00% |
| Huang 2022 (affected side) | 2 | -1a | 0 | 0 | -1c | -1d | very low | MD=-1.11 | [-2.31, 0.10] | 0.00% |
| Huang 2022 (unaffected side) | 4 | -1a | -1b | 0 | -1c | -1d | very low | MD=7.45 | [6.32, 8.57] | 62.00% |
| Huang 2022 | 6 | -1a | -2b | 0 | 0 | -1d | very low | MD=3.46 | [2.63, 4.28] | 96.00% |
| Zhang 2017b (affected side) | 4 | -1a | -2b | 0 | 0 | 0 | very low | SMD=-1.00 | [-1.90, -0.11] | 79.00% |
| Zhang 2017b (unaffected side) | 6 | -1a | -1b | 0 | 0 | 0 | low | SMD=0.57 | [0.04, 1.10] | 56.00% |
| ⑬Stroke severity |  |  |  |  |  |  |  |  |  |  |
| He 2020 | 7 | -1a | -1b | 0 | 0 | 0 | low | SMD=-0.555 | [-0.81, -0.30] | 0.00% |
| Tian 2011 | 2 | 0 | -2b | 0 | 0 | -1d | low | WMD=-0.89 | [-1.98, 0.19] | 75.10% |
| Li 2024 | 6 | -1a | -1b | 0 | 0 | -1d | very low | SMD=0.48 | [0.04, 0.93] | 69.00% |

Note: 0: not downgraded, -1: Decline one level, -2: Decline two levels. A is the specific randomization method, allocation concealment scheme or blindness not reported, included limitations in the original study design; b is small study credible interval overlap, large I2 value, large heterogeneity; 50% <I2 <75%, 75% <I2 c is small sample size, wide credible interval; d is few studies, possible publication bias; funnel map asymmetry. MD: mean difference, SMD: standardized mean difference, Hedges' g: SMD bias correction calculation, CI: confidence interval. NR: Not reported.
